# The Impact of Adverse Events in Transbronchial Lung Cryobiopsy on Histopathological Diagnosis

**DOI:** 10.3390/jcm14030731

**Published:** 2025-01-23

**Authors:** Kenji Tsumura, Shushi Umemoto, Yoshiaki Zaizen, Goushi Matama, Hidenobu Ishii, Sakiko Sumita, Yousuke Mitsui, Yutaka Ichikawa, Kazuhiro Tabata, Masaki Okamoto, Masaki Tominaga, Jun Akiba, Junya Fukuoka, Tomoaki Hoshino

**Affiliations:** 1 Division of Respirology, Neurology and Rheumatology, Department of Medicine, Kurume University School of Medicine, 67 Asahi-machi, Kurume 830-0011, Japan; tsumura_kenji@kurume-u.ac.jp (K.T.); umemoto_shuushi@kurume-u.ac.jp (S.U.); matama_goushi@med.kurume-u.ac.jp (G.M.); ishii_hidenobu@med.kurume-u.ac.jp (H.I.); sumita_sakiko@kurume-u.ac.jp (S.S.); mitsui_yousuke@kurume-u.ac.jp (Y.M.); ichikawa_yutaka@med.kurume-u.ac.jp (Y.I.); okamoto_masaki@med.kurume-u.ac.jp (M.O.); tominaga_masaki@med.kurume-u.ac.jp (M.T.); hoshino@med.kurume-u.ac.jp (T.H.); 2 Department of Pathology Informatics, Nagasaki University Graduate School of Biomedical Sciences, 1-7-1 Sakamoto, Nagasaki 852-8501, Japan; fukuokaj@nagasaki-u.ac.jp; 3 Department of Pathology, Kagoshima University Graduate School of Medical and Dental Sciences, 8-35-1 Sakuragaoka, Kagoshima 890-8544, Japan; tabatak.kufm@gmail.com; 4 Department of Respirology and Clinical Research Center, National Hospital Organization Kyushu Medical Center, 1-8-1 Jigyouhama, Chuo-ku, Fukuoka 810-8563, Japan; 5 Department of Community Medicine, Kurume University School of Medicine, 67 Asahi-machi, Kurume 830-0011, Japan; 6 Department of Pathology, Kurume University School of Medicine, 67 Asahi-machi, Kurume 830-0011, Japan; akiba@kurume-u.ac.jp

**Keywords:** pathology, interstitial lung disease, idiopathic pulmonary fibrosis, hemorrhage, safety, transbronchial cryobiopsy, complications, confidence level

## Abstract

**Background**: Transbronchial lung cryobiopsy (TBLC) has a high incidence of adverse events. This study aimed to investigate the relationship between the occurrence of these events and the condition of the pathology samples or pathological diagnosis in TBLC. **Methods**: We studied 102 patients who underwent TBLC for the diagnosis of interstitial lung disease. We analyzed the association between the condition or diagnosis of pathology samples and the occurrence of TBLC-related adverse events, including hemorrhage, pneumothorax, and acute exacerbation of interstitial lung disease. **Results**: The adverse events occurred in 19 patients (18.6%), of which hemorrhage was the most common (14 patients, 13.7%). The patients who experienced adverse events, especially hemorrhage, were less likely to have successful sampling with TBLC and showed lower diagnostic confidence in the pathology results. The diagnostic confidence was level A in 50 cases (49.0%) and level C in 23 cases (22.6%). TBLC-related adverse events, including hemorrhage, were significantly more common in patients with lower pathological confidence levels. **Conclusions**: TBLC-related adverse events, particularly hemorrhage, can lead to fewer successful samples and lower levels of diagnostic confidence.

## 1. Introduction

Transbronchial lung cryobiopsy (TBLC) has recently been reported to be useful in interstitial lung disease (ILD) as an alternative to surgical lung biopsy (SLB) [1,2,3]. Recently, we have widely used TBLC in the pathological diagnosis of ILD, showing a diagnostic yield of approximately 80% [2,4,5]. However, the pathological diagnosis is not always consistent between TBLC and SLB when performed on the same patient [6,7]. In the COLDICE study, which prospectively compared TBLC and SLB in 65 patients, factors contributing to the discordance between the two methods included age, family history of ILD, asymmetrical radiological findings, and the number of TBLC samples [8]. However, the size of the TBLC sample, TBLC freezing time, and the presence of pleura did not influence the discordance between TBLC and SLB [7]. We have previously reported that the pathological findings of TBLC and SLB are consistent with findings suggestive of usual interstitial pneumonia, but not with other findings [9]. Furthermore, we reported that the frequency of discrepancies in the pathological diagnosis of TBLC and SLB varies according to the disease [10].

The quality of pathology samples obtained via TBLC may contribute to the differences in assessment compared to SLB. Kuse et al. evaluated the sample quality of pathology and confidence level of pathological diagnoses [11]. They showed that, in cases where the quality of the pathology specimens was low, the diagnostic yield was low. No serious adverse events occurred in this study; however, many other studies have reported a higher frequency of adverse events with TBLC than with transbronchial lung biopsy (TBLB) or SLB. According to the official guidelines from the American Thoracic Society, the European Respiratory Society, the Japanese Respiratory Society, and the Latin American Thoracic Association (ALAT) for the diagnosis of idiopathic pulmonary fibrosis (IPF), the rate of adverse events associated with TBLC is reported to be 5.2% for hemorrhage and 16.5% for pneumothorax [2]. Another review article reported that the frequency of adverse events in TBLC was 30% for hemorrhage and 8% for pneumothorax [12]. The incidence of such adverse events with TBLC may also affect sampling. However, only a few reports examined the relationship between adverse events in TBLC and tissue sample quality or the accuracy of pathological diagnosis.

In this study, we investigated the impact of TBLC procedures and complications on the quality of pathology samples and the accuracy of pathological diagnoses.

## 2. Materials and Methods

### 2.1. Study Subjects

In conducting this study, we adhered to the tenets of the Declaration of Helsinki. We also sought and received approval from the Local Ethics Committee of Kurume University (No. 22140, 30 September 2022) to conduct this study. Informed consent was acquired from all the patients with an opt-out methodology.

This study was conducted using a dataset previously published by our group [13]. This retrospective observational study investigated 102 patients who underwent TBLC at our institution between April 2020 and September 2022. All patients underwent TBLC to diagnose diffuse lung disease, with a focus on ILD. We collected the medical information for these 102 patients, including patient characteristics and pulmonary function test results at the time of diagnosis. We also gathered information from the medical records on the biopsy procedures, including examination duration, sedation amount, and incidence of adverse events (hemorrhage, pneumothorax, and acute exacerbation of ILD) of TBLC.

### 2.2. The Procedure for Performing TBLC and Methods for Managing Adverse Events

TBLC was performed using a BF-1TQ290 bronchoscope or BF-1TH1200 bronchoscope (Olympus Corporation, Tokyo, Japan) and a 1.9 mm cryoprobe (Erbe Elektromedizin, Tübingen, Germany). We planned to obtain two or three specimens in every case. All patients underwent intubation with a flexible endotracheal tube and were maintained on spontaneous respiration under midazolam and fentanyl sedation, which is the standard protocol used in Japan. Pulse oxygen saturation, blood pressure, and electrocardiography were continuously monitored throughout the examination. The cryoprobe was inserted through the working channel of a flexible bronchoscope, placed 1 cm beneath the pleura under fluoroscopy, and activated for 6 to 7 s. We used a Fogarty catheter^TM^ (E-080-4F; Edwards Lifesciences, Irvine, CA, USA) for hemostasis, which was inflated after TBLC in all patients. About 3 to 4 min later, we deflated the balloon and checked for adverse hemorrhage events. Hemostatic procedures were carried out based on the severity of the hemorrhage, which included additional ballooning, intrabronchial adrenaline injections, and/or the administration of intravenous carbazochrome or tranexamic acid. Even if hemostasis was stopped with ballooning alone, further sampling with TBLC was aborted if the degree of hemorrhage was considered severe. In cases of pneumothorax, mild cases were monitored, while moderate or severe cases required thoracic drainage.

### 2.3. TBLC Adverse Events

Adverse events of hemorrhage were defined as cases requiring additional transbronchial or transvenous drug administration beyond ballooning or cases where TBLC sampling was discontinued before obtaining the adequate number of samples due to hemorrhage. Hemorrhage was classified as mild if hemorrhage could be controlled by ballooning alone, but the biopsy was discontinued due to hemorrhage; it was classified as moderate if epinephrine was administered into the bronchus and/or carbazochrome or tranexamic acid was injected into a vein. Severe adverse events of hemorrhage, needing surgical procedures, administration of blood, or admission to the intensive care unit, were not recorded in this study. Pneumothorax was considered a mild adverse event if it improved only with follow-up and was considered a moderate adverse event if thoracic drainage was required. No cases of pneumothorax requiring bullectomy were observed. Acute exacerbation of ILD was regarded as an adverse event related to TBLC if it occurred within 30 days of examination.

### 2.4. Pathological Diagnosis

Pathological diagnoses were made by three respiratory pathologists (Y.Z., K.T., and J.F.). Tissues obtained by TBLC were stained with hematoxylin and eosin and Elastica van Gieson, and whole-slide images (WSI) were obtained using a NanoZoomer S360 (Hamamatsu Photonics K.K., Shizuoka, Japan) at 200× magnification. The maximum length of the pathology samples was measured using WSI. The diagnostic confidence of pathology and sample quality scores were also evaluated using the method reported by Kuse et al. [11]. The quality score was defined as follows: Grade A specimens had sufficient lung tissue, including the acinus or some subacinus, bronchioles, and veins. Grade B specimens had a moderate amount of lung tissue between grades A and C. Grade C specimens were not easy to evaluate due to the presence of the bronchus alone or small amounts of alveoli. We defined the confidence level of the pathology as follows: level A specimens were suitable for establishing a definite pathological diagnosis; level B specimens are suggestive of a probable diagnosis; level C specimens only described a few findings, making it difficult to perform a pathological diagnosis.

**Figure 1:**
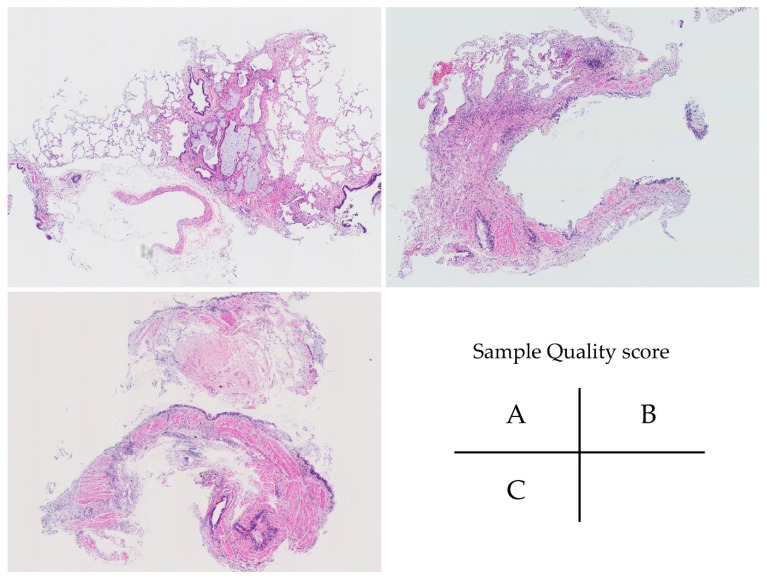
The samples of the quality score. Grade A specimens had sufficient lung tissue, including the acinus or some subacinus, bronchioles, and veins. Grade B specimens had a moderate amount of lung tissue between grades A and C. Grade C specimens were difficult to evaluate because of the presence of the bronchus alone or small amounts of alveoli.

The samples of the quality score are shown in Figure 1.

### 2.5. Statistical Analysis

In the present study, the participants were divided into two or three groups according to the presence or absence of adverse events due to TBLC and the confidence level of the pathology from TBLC. Their characteristics and evaluation of pathology samples were compared. Most numerical data are presented as median values with 25–75% of the interquartile range. For some numerical data, the mean values and standard deviations are provided. We analyzed the statistical significance of the differences between the two groups using the Fisher’s exact test or Wilcoxon rank–sum test. Statistical significance was set at *p* < 0.05, and all statistical analyses were conducted using the JMP 16.0 (SAS Institute, Cary, NC, USA).

## 3. Results

### 3.1. Patient Characteristics

The characteristics of the 102 patients included in the current study are presented in Table 1. The median age was 69 (60–75) years, and 56.9% of the patients were male. Adverse events occurred in 19 patients (18.6%), of whom 14 (13.7%) had a hemorrhage. There were nine cases in which the planned number of samples was not taken due to adverse events. Acute exacerbation of ILD happened in one patient (1.0%) who died because of this adverse event.

The pathological and MDD diagnoses are shown in Table 2. There were usual interstitial pneumonia (UIP) patterns in 38 patients (37.3%), nonspecific interstitial pneumonia (NSIP) patterns in 21 patients (20.6%), and organizing pneumonia (OP) patterns and acute lung injury in seven cases (6.9%) each. Pathological diagnosis could not be made in 14 patients (13.7%), and the diagnostic yield for TBLC in this study was 86.3%. The average number of specimens sampled by TBLC was 1.98, and the median maximum length among the samples was 7.0 mm. The sample quality at TBLC was favorable (score A) in 66 cases (64.7%), and in 17 cases (16.7%), the specimens were difficult to diagnose (score C). The pathological diagnostic confidence was good in 50 cases (49.0%, level A) and low or absent in 23 cases (22.5%, level C).

### 3.2. Adverse Event of TBLC

Table 3 presents a comparison of patient characteristics based on the presence or absence of adverse events. No correlation was observed between the occurrence of adverse events and age, sex, pathology pattern, or pulmonary function test results. Patients who experienced adverse events received additional sedation during the examination (2.89 mg vs. 2.10 mg, *p* = 0.010) were less likely to have successful TBLC sampling (1.63 vs. 2.06, *p* = 0.002) and had lower diagnostic confidence in the pathology (confidence level A: 21.1% vs. 55.4%, *p* = 0.006).

In the current study, the most frequent adverse event was hemorrhage. Consequently, we analyzed only the cases of hemorrhage-related adverse events. A comparison of patient characteristics according to the hemorrhage adverse events is shown in Table 4. Similar to all adverse events, additional sedation during the examination (3.14 mg vs. 2.10 mg, *p* = 0.004), number of samples (1.57 vs. 2.05, *p* = 0.002), and pathological diagnosis confidence level (confidence level A: 14.3% vs. 54.6%, *p* = 0.005) were correlated with the occurrence of hemorrhage.

### 3.3. Factors Affecting the Diagnostic Confidence Level of TBLC

Additionally, we investigated patient characteristics and the occurrence of adverse events in the three groups of patients according to the confidence levels of the pathological diagnoses with TBLC. The correlations between pathological confidence levels and patient characteristics are presented in Table 5. The diagnostic confidence was level A in 50 cases (49.0%), level B in 29 cases (28.4%), and level C in 23 cases (22.6%). A smaller number of successful TBLC samples and lower sample quality were associated with lower confidence levels in the pathology diagnosis (*p* = 0.001 and *p* < 0.001, respectively). Patient backgrounds, including age and sex, as well as pulmonary function test results, did not affect the confidence levels of pathology. Pathological patterns, TBLC examination time, and additional sedation dosage did not correlate with confidence levels. Adverse events, including hemorrhage, were significantly more frequent in patients with lower pathological confidence (*p* = 0.006, 0.008).

## 4. Discussion

In the present study, we have shown that there is a correlation between the quality of the pathology specimens in TBLC, pathological diagnosis, and the occurrence of adverse events in TBLC procedures. Diagnosis of ILD often requires histopathological examination, except in typical cases of IPF or hypersensitivity pneumonitis [3,14]. In the COLDICE study, TBLC was reported to have a high diagnostic concordance with SLB, the gold standard for pathological diagnosis in ILD [8]. However, other studies have indicated lower concordance between TBLC and SLB [6]. The COLDICE study also reported very high diagnostic concordance of pathology between TBLC and SLB in cases with high confidence levels of diagnosis [8]. To ensure an accurate multidisciplinary discussion (MDD) diagnosis using TBLC, it is necessary to maintain high specimen quality and diagnostic accuracy [11]. Our study demonstrated a correlation between TBLC-related adverse events and the accuracy of the pathological diagnosis of TBLC specimens. We demonstrated that effective management of TBLC-related adverse events is essential for obtaining high-quality samples and achieving high diagnostic confidence, which in turn reduces discordance and supports an accurate MDD diagnosis. To the best of our knowledge, no previous studies have reported the pathology and adverse events associated with TBLC.

In the current study, hemorrhage particularly affected the level of pathological diagnostic confidence among the adverse events. We consider that the decrease in the number of samplings due to adverse hemorrhage events led to the lower pathological diagnostic confidence level. In the present study, TBLC was sometimes discontinued without obtaining the planned number of biopsies due to hemorrhage. Such limited sample collections could pose a risk for inadequate pathological diagnosis. Ravaglia et al. reported that TBLC from one lung segment had a lower diagnostic yield than TBLC from two lung segments. [15]. If TBLC is halted before reaching the planned number of biopsies, it may result in only a single biopsy from one lung segment, potentially reducing diagnostic accuracy. The COLDICE study reported that factors related to discordance between TBLC and SLB pathology included the number of TBLC samples: 25% for three pieces, 55% for four pieces, and 80% for five pieces [7]. Therefore, it is important to biopsy additional samplings. Considering the results of our study and others, reducing adverse events, including hemorrhage, in TBLC may lead to secure sampling of specimens, which may contribute to improving the accuracy of pathological diagnosis.

To reduce adverse hemorrhagic events, it is essential to understand their causes and evaluate risk factors associated with TBLC. We believe that central biopsies, performed to avoid pneumothorax, are a primary cause of hemorrhage compared to TBLB. Generally, biopsies too close to the middle third of the lung increase the risk of severe hemorrhage [16]; therefore, care should be taken when taking a biopsy from the peripheral side of the lung. Regarding the risk of hemorrhage, we reported that obesity is a contributing factor in our study [13]. Obesity is thought to decrease lung compliance and increase pulmonary blood flow due to fat deposits in the thoracic cavity. Low pulmonary function has also been reported as a risk factor for adverse events [17]. Additionally, the incidence of hemorrhage possibly increased in patients with abnormal coagulation parameters and those using clopidogrel or other new antiplatelet drugs [18]. Pulmonary hypertension has also been reported as a risk factor for hemorrhage in TBLC [19]. For TBLC in such high-risk patients, we may need to be more cautious in our testing practices or even consider switching to other means of diagnosis, such as SLB.

Finally, we found that the additional use of midazolam during the examination may be linked to decreased diagnostic accuracy. We think this is because of the inadequate sedation. Inadequate sedation prior to the induction of the examination may lead to the need for additional sedation, prolonging the examination time and decreasing the number of specimens collected. In conclusion, our findings show that adequate sedation with appropriate sedatives prior to examination to prevent adverse events, such as hemorrhage, may contribute to a more accurate diagnosis of pathology. Recently, Lachowicz et al. showed in their systematic review article that general anesthesia was associated with a higher diagnostic yield for TBLC [20], which is consistent with our study.

In this study, we found the data to be reliable concerning the correlation between adverse events and pathological diagnoses. Specifically, 64.7% of the pathological specimens were of good quality, and 49.0% of the pathological diagnoses were performed with a high degree of certainty. Although the quality of the specimens was slightly lower than that of the previous report [11], the pathological diagnostic confidence was comparable. As for test-related adverse events, hemorrhage occurred in 13.7%, pneumothorax in 4.9%, and acute exacerbation of interstitial pneumonia in 1.0% of patients, showing no significant deviation from previously reported results [2,12].

This study had several limitations. First, this study was conducted in a single center and did not include many patients. Second, there were a small number of occurrences of pneumothorax or acute exacerbation of ILD in this study. Third, the management of adverse events, particularly hemorrhage, was entirely at the discretion of the examiner. We did not have a set protocol for discontinuing the examination or administering hemostatic agents. This indicates the existence of examiner bias. However, since a single physician (GM) oversaw and conducted all examinations in this study, we believe that examiner bias was minimal. Future research should focus on conducting multicenter studies with larger patient populations to improve generalizability and enhance statistical power. Furthermore, we believe that TBLC procedures need to be standardized and then validated in a multicenter study to evaluate whether the accuracy of the pathological diagnosis correlates with adverse events. This will provide a more comprehensive understanding of TBLC-related adverse events across different settings.

## 5. Conclusions

The present study indicated that TBLC-related adverse events, particularly hemorrhage, can lead to fewer successful samples and lower levels of diagnostic confidence.

## Figures and Tables

**Table 1:** Patient characteristics.

**No. of Patients**	102	All Adverse Events	19 (18.6%)
**Sex (Male)**	58 (56.9%)	Hemorrhage	14 (13.7%)
**Age (years)**	69 (60–75)	Mild	6 (5.9%)
**BMI (kg/m^2^)**	24.0 (20.4–26.1)	Moderate	8 (7.8%)
**%FVC (%)**	84.3 (68.5–99.1)	Pneumothorax	5 (4.9%)
**%D_LCO_ (%)**	74.9 (60.9–96.0)	Mild	2 (2.0%)
**FEV1 (L)**	1.94 (1.54–2.50)	Moderate	3 (2.9%)
**Examination time (min)**	29 (25–35)	Acute Exacerbation of ILD	1 (1.0%)
**Midazolam dosage**	5 (4–6)		

BMI, body mass index; D_LCO_, diffusing capacity of the lung for carbon monoxide; FEV1, forced expiratory volume in 1 s; FVC, forced vital capacity; ILD, interstitial lung disease.

**Table 2:** Pathological evaluation.

Pathological Diagnosis		MDD Diagnosis	
** UIP pattern**	38 (37.3%)	IPF	17 (16.7%)
** NSIP pattern**	21 (20.6%)	Other IIPs	6 (5.9%)
** OP pattern**	7 (6.9%)	CTD	21 (20.6%)
** Acute lung injury**	7 (6.9%)	HP	19 (18.6%)
** Other ILD**	15 (14.7%)	Unclassifiable ILD	13 (12.7%)
** Non diagnostic**	14 (13.7%)		
**No. of samplings (average)**	1.98 ± 0.54		
**Maximum length of the samples (mm)**	7.0 (6.3–7.9)		
**Sample quality score** **(A/B/C)**	66/19/17 (64.7%/18.6%/16.7%)		
**Confidence level** **(A/B/C)**	50/29/23 (49.0%/28.4%/22.5%)		

ILD, interstitial lung disease; MDD, multidisciplinary discussion; NSIP, nonspecific interstitial pneumonia; OP, organizing pneumonia; UIP, usual interstitial pneumonia.

**Table 3:** A comparison of patient characteristics across all adverse events is provided.

	With Any AE	Without All AE	*p* Value
**No. of patients**	19 (18.6%)	83 (81.3%)	
**Sex (Male)**	14 (73.7%)	44 (53.0%)	0.127
**Age (years)**	64 (52–77)	69 (62–75)	0.402
**BMI (kg/m^2^)**	24.1 (21.1–30.6)	24.0 (20.0–25.9)	0.221
**%FVC (%)**	84.8 (72.2–97.5)	83.7 (67.7–100.0)	0.937
**%D_LCO_ (%)**	73.7 (63.2–88.3)	77.8 (59.3–97.1)	0.623
**FEV1 (L)**	2.29 (1.75–2.49)	1.86 (1.49–2.50)	0.150
**UIP pattern**	8 (42.1%)	30 (36.1%)	0.583
**Examination time (min)**	35 (25–40)	28 (25–35)	0.059
**Additional Midazolam during the examination (mg, average)**	2.89 ± 1.37	2.10 ± 1.34	0.010
**No. of samplings (average)**	1.63 ± 0.68	2.06 ± 0.48	0.002
**Maximum length of the samples (mm)**	6.7 (5.2–7.7)	7.0 (6.4–8.0)	0.078
**Sample quality score A/C**	10 (52.6%)/5 (26.3%)	56 (67.5%)/12 (14.5%)	0.345
**Confidence level A/C**	4 (21.1%)/9 (47.4%)	46 (55.4%)/14 (16.9%)	0.006

AE, adverse event; BMI, body mass index; D_LCO_, diffusing capacity of the lung for carbon monoxide; FEV1, forced expiratory volume in 1 s; FVC, forced vital capacity; UIP, usual interstitial pneumonia.

**Table 4:** A comparison of patient characteristics for adverse event of hemorrhage.

	With Hemorrhage	Without Hemorrhage	*p* Value
**No. of patients**	14 (13.7%)	88 (86.3%)	
**Sex (Male)**	10 (71.4%)	48 (54.6%)	0.264
**Age (years)**	62 (52–77)	70 (62–75)	0.439
**BMI (kg/m^2^)**	23.7 (21.2–35.4)	24.0 (20.0–25.9)	0.097
**%FVC (%)**	87.3 (70.4–99.4)	83.5 (68.1–99.1)	0.695
**%D_LCO_ (%)**	73.7 (65.3–92.6)	76.2 (59.7–96.5)	0.903
**FEV1 (L)**	2.33 (1.76–2.50)	1.87 (1.49–2.50)	0.138
**UIP pattern**	7 (50.0%)	31 (35.2%)	0.347
**Examination time (min)**	35 (23–42)	28 (25–35)	0.155
**Additional Midazolam during the examination (mg, average)**	3.14 ± 1.23	2.10 ± 1.35	0.004
**No. of samplings (average)**	1.57 ± 0.76	2.05 ± 0.48	0.002
**Maximum length of the samples (mm)**	7.3 (5.2–7.8)	6.9 (6.4–8.0)	0.422
**Sample quality score A/C**	7 (50.0%)/4 (28.6%)	59 (67.1%)/13 (14.8%)	0.330
**Confidence level A/C**	2 (14.3%)/7 (50.0%)	48 (54.6%)/16 (18.2%)	0.005

BMI, body mass index; D_LCO_, diffusing capacity of the lung for carbon monoxide; FEV1, forced expiratory volume in 1 s; FVC, forced vital capacity; UIP, usual interstitial pneumonia.

**Table 5:** Patient characteristics affecting diagnostic confidence level of pathology with TBLC.

	Level A	Level B	Level C	*p* Value
**No. of patients**	50 (49.0%)	29 (28.4%)	23 (22.6%)	
**Sex (Male)**	23 (46.0%)	20 (69.0%)	15 (65.2%)	0.091
**Age (years)**	67 (60–74)	71 (61–77)	71 (52–75)	0.675
**BMI (kg/m^2^)**	23.7 (19.5–25.8)	23.9 (20.3–26.5)	24.8 (22.2–26.1)	0.334
**%FVC (%)**	83.5 (72.6–100.5)	80.6 (65.5–97.0)	87.1 (64.2–102.2)	0.875
**%D_LCO_ (%)**	80.5 (63.5–98.0)	74.8 (56.9–100.4)	68.7 (58.9–83.9)	0.174
**FEV1 (L)**	1.91 (1.53–2.51)	2.09 (1.46–2.50)	1.83 (1.56–2.50)	0.991
**UIP pattern**	17 (34.0%)	16 (55.2%)	5 (21.7%)	0.165
**Examination time (min)**	28 (24–35)	29 (25–38)	34 (25–38)	0.349
**Additional Midazolam during the examination (mg, average)**	2.34 ± 1.32	1.93 ±1.53	2.43 ±1.27	0.221
**No. of samplings (average)**	2.06 ± 0.47	2.13 ± 0.58	1.61 ± 0.50	0.001
**Maximum length of the samples (mm)**	7.0 (6.4–8.1)	7.2 (6.4–8.0)	6.6 (5.2–7.9)	0.307
**Sample quality score A/C**	42 (84.0%)/0 (0.0%)	21 (72.4%)/3 (10.3%)	3 (13.0%)/14 (60.9%)	<0.001
**All adverse events**	4 (8.0%)	6 (20.7%)	9 (39.1%)	0.006
**Hemorrhage adverse events**	2 (4.0%)	5 (17.2%)	7 (30.4%)	0.008

BMI, body mass index; D_LCO_, diffusing capacity of the lung for carbon monoxide; FEV1, forced expiratory volume in 1 s; FVC, forced vital capacity; UIP, usual interstitial pneumonia.

## Data Availability

The datasets used and/or analysed during the current study available from the corresponding author on reasonable request.
